# Opium in the Treatment of Chronic Ulcers

**Published:** 1855-03

**Authors:** 


Opium in the Treatment of Chronic Ulcers. By Mr. Skey.—Opium produces a certain effect on the chronic and callous ulcer; if it answers here, it will in other diseases, having the special characters of the chronic ulcer. The pharmacologist tells us it is a stimulant and sedative. What do you understand by the stimulating properties of opium? I should be at a loss to call to mind any cases in which it is given as a stimulant, unless in such as these. In nineteen out of twenty instances it is employed as a sedative, as, for example, in relaxed bowels, and to promote sleep. I can show you it possesses other properties quite equal to these; it prevents sleep sometimes; when taken continually it tranquilizes, but it will not always produce sleep. What is the action of opium to which I allude? Whether it acts on the center or the periphery, whether on the heart or capillaries, I do not know. But this I do know, that it promotes a healthy action on the capillaries of the body, and in cases of chronic ulcer, which I select as an example, you will have a local action set up, which will heal a wound,—will set up a healthy granulating surface, and completely heal it up in a short time, when every other method has failed to do so. What is the condition and character of a chronic ulcer? A chronic ulcer is an excavation, for the most part, affecting the limbs of old people, generally the inner side of the lower limbs; two processes are going on, the formation of the excavation, and the formation of a ring of organized lymph which encircles the excavation, thus hollowing it to a considerable depth, and its surface is covered with unhealthy lymph. This is common to all of them; the elevation of the margins is not a specific manifestation, as it is common to other forms of ulcer. Under the microscope there is not a granulation to be seen there is an ichorous discharge, with an offensive fœtor arising from it, and yet there is no sensibility, no annoyance nor suffering to the patient, it goes on for year after year without any change; you may strap it, there is no change produced. I give opium, what does it do? It seems to start the capillary systim all over the body, particularly in the lower extremities; it produces a genial glow, which may be compared to that of a cold bath, but not from such as is ordinarily taken; go into a warm bath, with a meal in the stomach, with or without perspiration, it makes no difference; well, then, the glow is produced by the opium, and healthy granulations over the whole surface of the ulcer will become apparent in a week. What is the sum of the actions of opium? You will say it is unhealthy. It is not; it promotes warmth, the pouring out of healthy secretions, it produces healthy integument, healthy granulations, it absorbs the unsound around it, and, while the granulations are coming up the elevations are going down. That cannot be considered unhealthy which promotes health.—*Medical Press.*

